# Psychometric validation and determination of minimal clinically important differences for the strengths and difficulties questionnaire in adolescents with myopia

**DOI:** 10.3389/fpubh.2025.1730452

**Published:** 2026-01-06

**Authors:** Ruosong Yang, Hongpo Yin, Yuanyuan Hu, Shijie Yu, Ronghua Lai, Wei Sun, Hongsheng Bi, Jianfeng Wu

**Affiliations:** 1 Medical College of Optometry and Ophthalmology, Shandong University of Traditional Chinese Medicine, Jinan, Shandong, China; 2 Affiliated Eye Hospital of Shandong University of Traditional Chinese Medicine, Jinan, Shandong, China; 3 Shandong Provincial Key Laboratory of Integrated Traditional Chinese and Western Medicine for Prevention and Therapy of Ocular Diseases, Shandong Provincial Clinical Medical Research Center of Optometry and Adolescent Low Vision Prevention and Control, Shandong Engineering Technology Research Center of Visual Intelligence, Jinan, Shandong, China

**Keywords:** myopia, mental health, strengths and difficulties questionnaire, psychometrics validation, minimal clinically important difference

## Abstract

**Background:**

Myopic adolescents face multiple psychological challenges, including anxiety, depression, and social isolation, necessitating multidimensional assessment. One such assessment tool is the Strengths and Difficulties Questionnaire (SDQ), though it has not yet been validated in this specific population. This study aimed to evaluate the reliability, validity, responsiveness, and the minimal clinically important difference (MCID) of the SDQ in this specific population.

**Methods:**

Following the consensus-based standards for the selection of health measurement instruments (COSMIN) guidelines, we conducted a psychometric evaluation combining cross-sectional and longitudinal designs among 307 adolescents with myopia (including newly diagnosed and follow-up cases). Baseline assessments evaluated feasibility, reliability (internal consistency and test–retest stability), and validity (construct and discriminant validity). Follow-up surveys after myopia correction were used to assess responsiveness and establish the MCID using anchor-based methods.

**Results:**

The SDQ demonstrated acceptable internal consistency (Cronbach’s *α* = 0.71) and good test–retest reliability (ICC = 0.73). Construct validity was supported by strong correlations between problem-related subscales (peer problems, hyperactivity/inattention, conduct problems, emotional problems) and difficulty scores (*r* = 0.51–0.72, *p* < 0.001), with moderate-to-strong correlations for total SDQ scores (*r* = 0.36–0.75, *p* < 0.001). Discriminant validity was confirmed by significant differences in prosocial behavior (*p* < 0.01). While responsiveness was modest [effect size (ES) = −0.26, standardized response mean (SRM) = −0.28], the MCID was determined to range between 2.00–2.38 points, with a threshold of 2 points recommended for group-level discrimination.

**Conclusion:**

The SDQ is a reliable and valid tool for assessing psychological well-being in adolescents with myopia. The established MCID of 2 points enhances its clinical utility for screening and monitoring mental health changes in this population.

## 1 Introduction

Children with myopia are prone to psychological problems than their emmetropic peers. This population faces dual challenges of myopia progression and concurrent mental health issues (1). Myopia can alter brain pathways tied to emotions and motivation, acting like a persistent stressor that triggers anxiety (2). Early-stage blurry vision can cause anxiety symptoms (3), which may be exacerbated by optical correction—especially when corrected with glasses rather than contact lenses (4). Worsening myopia introduces additional stressors including parental pressure and physical discomfort, while social and familial factors often lead to avoidance of vision correction, accelerating both visual decline and psychological strain. Several factors related to children, society, and families lead kids with myopia to avoid vision correction (5–7).

Although most studies indicate poorer mental health in myopic populations (4, 8), methodological inconsistencies, including diverse assessment tools and sampling biases, have produced conflicting findings (9, 10), underscoring the need for validated screening instruments. Upon entering adolescence, individuals undergo significant neuroendocrine system remodeling, including heightened plasticity of the hypothalamic–pituitary–adrenal (HPA) axis and fluctuating sex hormone levels. These changes themselves have been demonstrated to lead to heightened emotional reactivity and a predisposition toward generalized anxiety (11). During this critical developmental stage, myopia exerts a unique influence as a persistent external stressor by limiting visual function, altering physical appearance, and triggering social barriers. These effects compound with the inherent biopsychosocial changes of adolescence, collectively fostering distinct psychological vulnerability. Therefore, distinguishing between transient emotional fluctuations stemming from adolescent development and specific psychological issues arising from myopia and its psychosocial consequences is crucial during assessment. Without appropriate intervention, the latter are more likely to solidify into persistent psychological disorders (12). A recent large-scale study confirmed that myopic children were 3.60 times more likely to exhibit abnormal psychological problems than their non-myopic peers (13), highlighting a substantial added burden. This compelling evidence underscores the necessity of employing a validated, multidimensional tool like the SDQ to better understand and assess the psychological challenges associated with myopia in this age group.

Early identification of comorbid mental health issues in this population requires multidimensional assessment tools. Existing research mainly relies on scattered tools targeting isolated symptoms, without a systematic psychometric evaluation of this population, which limits the reliability of the analysis of psychological characteristics (Supplementary Table 1). Adolescents with myopia may experience overlapping psychological challenges including anxiety, depression, and social isolation, necessitating screening approaches that capture this complexity (14). The SDQ offers particular promise due to its multidimensional framework evaluating emotional symptoms, behavioral issues, hyperactivity, peer problems, and prosocial behavior (15, 16). The “Peer Relationship Problems” and “Emotional Symptoms” subscales align closely with the common psychosocial impacts of myopia, while the “Prosocial Behavior” subscale assesses positive social behaviors (17). Therefore, the SDQ can identify both universal developmental challenges and psychological distress specific to myopia. While several studies have evaluated the psychometric properties of the SDQ in different populations (18, 19), there is a gap in the current research on myopia. And clinical interpretation of scores remains limited without established thresholds for meaningful change. The minimum important change (MIC) can be used as one of the outcome measures (20). However, there is currently a lack of MCID values for the SDQ scale in any population.

The purpose of this study is to comprehensively assess the psychometric properties of the SDQ scale and to attempt to develop MCID. The objective is to validate the tool’s capability in early identification of psychological comorbidity and to optimize research methodologies and clinical intervention strategies for this vulnerable population.

## 2 Methods

### 2.1 Study design and participants

This study was conducted from December 2023 to June 2025 using purposive sampling. Participants aged 6–15 years were enrolled, with age parameters aligned to myopia onset peaks and SDQ validation norms. Inclusion required: (1) Cycloplegic spherical equivalent (SE) ≤ −0.50 D (21); (2) No ocular pathology (ophthalmologist-confirmed); (3) No concurrent research participation; (4) Written informed consent. Exclusion criteria: cognitive/communication impairments or systemic diseases. The sample size was estimated according to the COSMIN guidelines and the official SDQ scale manual (22, 23).

Participants were selected through a multi-center recruitment strategy: children newly diagnosed with myopia were registered at ophthalmology clinics, while children with pre-existing myopia were screened at ophthalmology hospitals and two schools. All participants and their guardians provided informed consent after institutional review board approval and received interview incentives. To ensure representativeness, recruitment was stratified according to the prevalence of current primary myopia correction methods (single-vision correction, myopia control glasses, and orthokeratology lenses). The questionnaire was developed in consultation with optometrists at the vision care center and validated through a one-week pilot survey prior to formal data collection. Trained researchers with a background in optometry (who had received training in interview techniques and signed confidentiality agreements) conducted face-to-face structured interviews, explaining the study protocol and follow-up schedule. Follow-up assessments were conducted online via the Wenjuanxing platform^1^: newly diagnosed children were surveyed 20–28 days after diagnosis, while children with recurrent myopia completed follow-up within 5–7 days.

### 2.2 Outcome measures

The measurements encompassed general information, the results of ophthalmological examinations, the global rating of change questionnaire (GRCQ), the EuroQol five-dimension questionnaire for youth (EQ-5D-Y), and the Chinese version of the SDQ scale. The self-designed general information and eye examination data included the following: age, gender, ethnicity, education level, body mass index (BMI), reason for outpatient visit, myopia correction method, length of time with myopia, self-assessed myopia control effect, and spherical equivalent refraction (SER) after pupil dilation.

The SDQ Scale is a brief psycho-behavioral screening tool used to assess emotional, behavioral, and social functioning in children and adolescents. It has five subscales with 25 entries, and each question is rated on three levels: not true, somewhat true, and certainly true (16). Access is available online for free.

The GRCQ comprises a single entry. The specific question adapted for use in this study was “After myopia correction treatment, how has your overall health changed from the last measurement?” The response options were derived from a 5-point Likert scale, ranging from “significantly improved” to “slightly worse” (24). This scale was used to assess the reactivity of the SDQ scale.

The EQ-5D-Y scale assesses five health dimensions (mobility, self-care, usual activities, pain/discomfort, anxiety/depression), with each dimension having three severity options (25). This scale uses only a single question on the emotional dimension and was used as an external anchor in the development of the MCID.

### 2.3 Patient or public contribution

Children with myopia who visited the optometry center, as well as optometrists from the ophthalmology hospital, participated in this study. Optometry experts contributed to the study’s overall design, including the questionnaire’s content and ophthalmic data. They also helped define the age range of participants and clarify the study’s objectives and research questions. During the pre-survey phase, myopic children and their parents who visited the hospital provided informed consent to participate. However, due to the incomplete questionnaire, this group was excluded from the formal study, though they provided valuable feedback for optimizing the final version of the questionnaire. Optometrists and interviewers communicated irregularly and adjusted the questionnaire based on feedback from the children and their parents or guardians. They removed certain privacy-related questions (e.g., annual household income) and optimized the wording of some content. After completing the questionnaire, optometrists immediately reviewed the content to ensure data completeness and accuracy. The guardians of the participants were contacted by phone to inform them of their children’s examination results and provide medical guidance based on the questionnaire results obtained. Optometrists introduced the study to patients during consultations to promote its dissemination. WS is an optometrist and a co-author of this paper, contributing to its preparation from the early drafts to the final version.

### 2.4 Ethics approval statement

The human participants involved in this study adhered to the provisions of the Declaration of Helsinki. This study was approved by the Medical Ethics Committee of Shandong University of Traditional Chinese Medicine Eye Hospital (HEC-KS-202305KY; HEC-HY-2024040KY).

### 2.5 Statistical analysis

All statistical analyses were conducted using SPSS v27.0.1.

Baseline characteristics were first described using descriptive statistics. Categorical variables were expressed as frequency and percentage (*n*, %), while continuous variables were presented as mean ± standard deviation. Intergroup comparisons were performed using chi-square tests or t-tests, with *p* < 0.05 indicating statistically significant differences.

The psychometric properties of the scale assessed (22): (1) feasibility via scale acceptance/completion rates/time; (2) reliability through test–retest reliability (Pearson correlations), intra-group consistency (ICCs), internal consistency (Cronbach’s *α*), and split-half reliability; (3) validity via content/structural validity (subscale correlations) and discriminant validity (Mann–Whitney U tests across myopia durations); (4) responsiveness using Wilcoxon signed-rank tests for baseline-followup SDQ score changes.

MCID values were derived via distributional (0.5 times SD and SEM) and anchor-based methods (EQ-5D-Y anxiety/depression domain with ‘slight change’ as threshold), then validated against Minimal Detectable Change (MDC) metrics—ratios >1 confirming true health status change over measurement error. Finally, the validity of the obtained MCID values was analyzed by calculating the ratio of MCID to MDC (ind) and MDC (group).

## 3 Results

**Figure 1:**
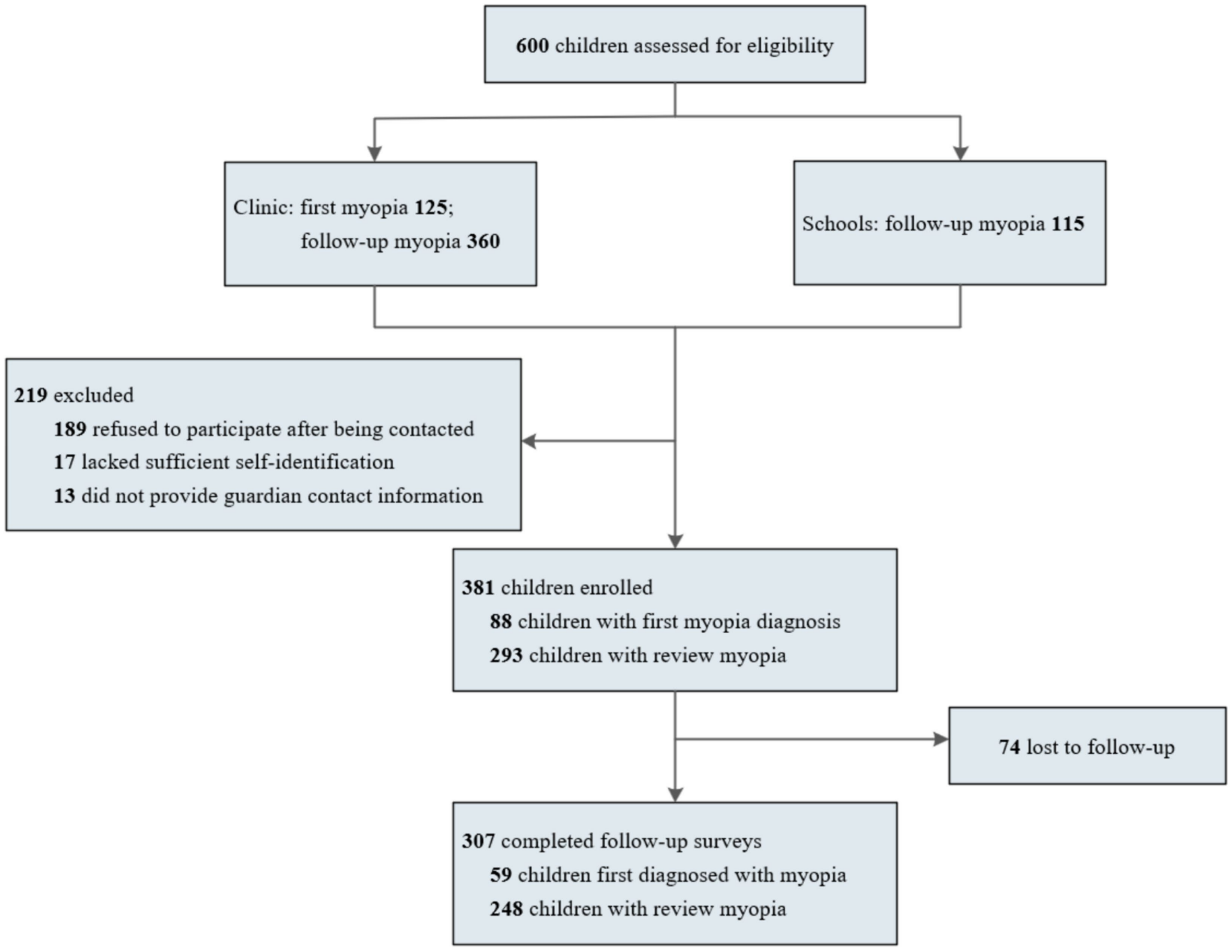
Flowchart of patient enrollment.

Between December 2023 and November 2024, 125 patients with myopia detected at initial examination and 360 children with myopia follow-up were invited to the clinic, and 115 myopic children were invited to participate in this investigation in two schools. Communication and screening were conducted, of which 381 children were considered eligible for the study. During the follow-up phase, 74 patients withdrew from the study. Therefore, a total of 293 children were included in the study (Figure 1).

Baseline patient characteristics are presented in Supplementary Table 2. Participants exhibited significant clinical heterogeneity, encompassing children with myopia of varying severity, disease progression stages, and management strategies. Significant differences existed between newly diagnosed myopic children (*n* = 88) and follow-up myopic children (*n* = 293) across multiple baseline characteristics. The proportion of primary and secondary school students in the newly diagnosed group (88.64% vs. 72.70%; *p* = 0.002) and the proportion with low body mass index (BMI) (63.64% vs. 44.37%; *p* = 0.005) were significantly higher than in the follow-up group. Furthermore, both groups exhibited significant differences in myopia-related clinical characteristics such as reason for visit, myopia type, management regimen, and self-reported control efficacy (all *p* < 0.001).

### 3.1 Feasibility analysis

The first survey was administered to myopic children and adolescents who signed informed consent during a face-to-face interview. The acceptance and completion rates of the SDQ scale were 100%, meeting the minimum requirement of 85% acceptance and completion rates. The recall rate of the follow-up scale was 80.58%. The shortest time to complete the SDQ scale by the study participants was 170 s, and the longest time was 277 s, with a mean time of 209.04 ± 23.16 s.

### 3.2 Reliability

**Figure 2:**
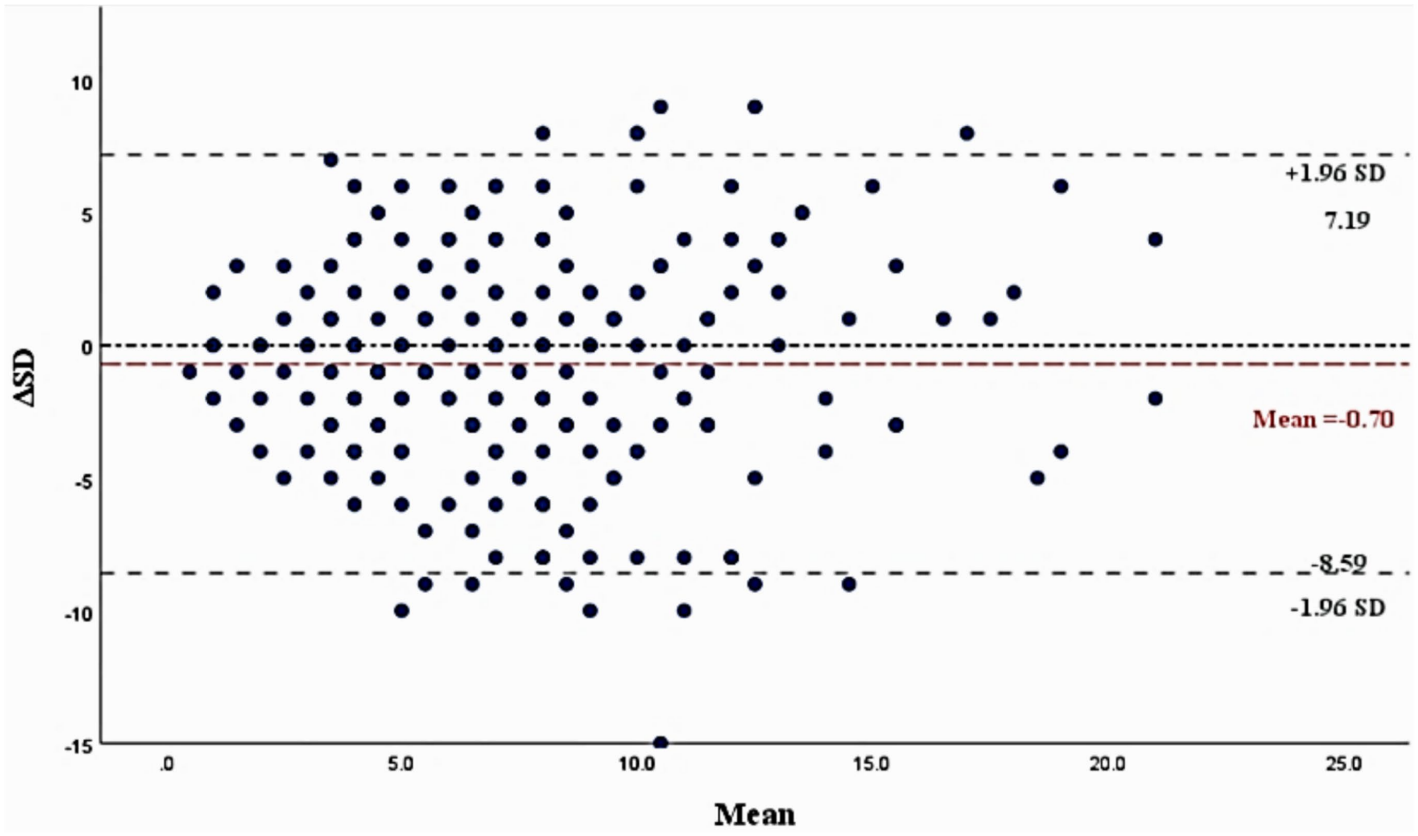
Bland–Altman plot of two-measure difficulty scores on the SDQ scale. ΔSD, The scoring difference between the two SDQ difficulty scores; Mean, The mean scores for both SDQ difficulty scores; 95% limits of agreement (95% LoA): −8.59 ~ 7.19.

The reliability analysis results indicate that the SDQ questionnaire demonstrates good measurement stability and consistency within the study population. The test–retest reliability of the questionnaire is satisfactory, with an intraclass correlation coefficient (ICC) of 0.73, exceeding the standard threshold of 0.7. The Pearson correlation coefficient between the two tests was 0.575, considered acceptable (26). From the Bland–Altman plot, the percentage of the difference between the total SDQ difficulty score ratings of the two measurements that were within its LoA was 94.35% (234/248), and based on the view that the difference between the measurement ratings was within its 95% LoA, the two-measurement consistency of the SDQ scale was considered fair (26) (Figure 2). However, the split-half reliability assessed using the Spearman-Brown coefficient was 0.532, below the 0.7 threshold, indicating poor internal consistency between the two halves of the scale. In contrast, internal consistency measured by Cronbach’s alpha was acceptable for both all items (*α* = 0.722) and the five factors plus total score (*α* = 0.708), with both values meeting the >0.7 criterion (27) (Table 1).

**Table 1:** Reliability analysis for the SDQ scale.

Reliability metric	Result	Criterion	Evaluation
Test–retest reliability			Good
ICC	0.730	>0.70	Good
Pearson correlation coefficient	0.575	>0.50	Acceptable
Split-half reliability	0.532	>0.70	Poor
Internal consistency (Cronbach’s *α*)			Acceptable
All items	0.722	>0.70	Acceptable
Five factors and total score	0.708	>0.70	Acceptable

### 3.3 Validity

The SDQ inventory demonstrated good content validity in this sample of myopic adolescents. Pearson correlation analysis revealed the strongest association between the total difficulty score and the behavioral problems dimension (*r* = 0.725, *p* < 0.001), followed by emotional symptoms (*r* = 0.716, *p* < 0.001) and hyperactivity/inattention (*r* = 0.713, *p* < 0.001), all showing strong correlations with the total difficulty score; The total difficulty score showed a moderate correlation with the peer relationship dimension (*r* = 0.512, *p* < 0.001). Meanwhile, the correlations among the dimensions (0.137–0.397) were significantly lower than their correlation with the total score. This pattern indicates that while the subscales collectively constitute the overall construct, they also measure relatively independent psychological characteristics, providing evidence for the scale’s discriminant validity (Table 2).

**Table 2:** The correlation analysis of the four SDQ difficulty subscales with difficulty scores.

SDQ scale domains	Peer relationship problems	Hyperactivity/inattention	Conduct problems	Emotional symptoms	Total difficulties score
Peer relationship problems	1.000				
Hyperactivity/inattention	0.137^*^	1.000			
Conduct problems	**0.217** ^***^	**0.373** ^***^	1.000		
Emotional symptoms	0.156^**^	**0.297** ^***^	**0.397** ^***^	1.000	
Total difficulties score	**0.512** ^***^	**0.713** ^***^	**0.725** ^***^	**0.716** ^***^	1.000

Bold values indicate statistically significant differences (*p* < 0.001). ^*^p < 0.05; ^**^p < 0.01; ^***^p < 0.001.

To evaluate the scale’s ability to distinguish different clinical subgroups, we conducted comparative analyses based on disease progression (initial diagnosis group versus follow-up group) (Table 3). Results showed that the follow-up group scored significantly higher than the initial diagnosis group on prosocial behavior (difference = 0.132, *p* = 0.024) and peer relationship problems (difference = 0.078, *p* = 0.031), indicating the scale effectively captures specific social functioning changes associated with long-term myopia. However, no significant differences were observed between groups in core psychological distress dimensions (hyperactivity/inattention, conduct problems, emotional symptoms) or total scores (all *p* > 0.05). This suggests these core psychological traits may be relatively stable and less influenced by the myopia course itself. These findings provide conditional support for the discriminant validity of the SDQ in this population.

**Table 3:** Comparison of SDQ scale difficulty scores in patients with different disease stages.

SDQ scale domains	Disease duration		*Z*	*p*
	=0	>0		
Prosocial behavior	0.088 ± 0.364	0.220 ± 0.579	−2.253	**0.024**
Peer relationship problems	0.062 ± 0.263	0.140 ± 0.377	−2.153	**0.031**
Hyperactivity/inattention	0.093 ± 0.356	0.110 ± 0.399	−0.245	0.806
Conduct problems	0.161 ± 0.479	0.150 ± 0.386	−0.508	0.611
Emotional symptoms	0.052 ± 0.284	0.060 ± 0.312	−0.163	0.870
Total difficulties score	0.078 ± 0.321	0.090 ± 0.321	−0.552	0.581
Total score	0.798 ± 0.788	0.790 ± 0.782	0.068	0.946

Myopia progression: 0 = initial diagnosis of myopia; > 0 = follow-up diagnosis of myopia. Bold values indicate statistically significant differences (*p* < 0.05).

To further examine the scale’s structure, we analyzed the full-scale correlation matrix including the “prosocial behavior” dimension (see Supplementary Table 3). The results revealed the scale’s anticipated theoretical structure: “Prosocial Behavior” showed significant negative correlations with all four difficulty subscales (r: −0.264 to −0.396, all *p* < 0.001). This aligns with the dimension’s definition as positive, protective behavior, demonstrating good discriminant validity. Furthermore, these findings reconfirmed the significant positive correlations within the difficulty dimensions.

### 3.4 Responsiveness

To assess the SDQ scale’s ability to detect clinically important changes, we conducted a comprehensive analysis using the Wilcoxon signed-rank test, effect size (ES), and standardized response mean (SRM) (Table 4). Results showed that the total difficulty score in the full sample decreased significantly by 1.186 points after intervention (*p* = 0.049), with both ES (−0.259) and SRM (−0.281) meeting the “small” effect size criterion. In subgroup analysis, the improvement group showed a significant decrease in difficulty scores (−1.053 points, *p* < 0.001), with ES and SRM also indicating a “small” effect size (−0.222 and −0.254, respectively). In contrast, the non-improvement group showed a non-significant change (−0.944 points, *p* = 0.096). Although their ES and SRM (−0.234 and −0.243, respectively) were comparable to the improvement group, they lacked statistical support. These findings consistently demonstrate that the SDQ effectively captures subtle changes in group-level mental health status resulting from myopia correction interventions, indicating adequate responsiveness (Table 4).

**Table 4:** Assessment of SDQ scale responsiveness and comparison of pre- and post-treatment scores according to GRCQ.

Responsiveness parameters	Total difficulties score		
	Full sample (59)	Improvement sample (38)	Unchanged sample (18)
Baseline score	8.220 ± 4.579	7.711 ± 4.741	9 ± 4.029
Follow-up score	7.034 ± 4.533	6.658 ± 3.744	8.056 ± 5.173
Rating difference	−1.186 ± 4.224	−1.053 ± 4.152	−0.944 ± 3.888
*p*	**0.049**	**<0.001**	0.096
ES	−0.259	−0.222	−0.234
SRM	−0.281	−0.254	−0.243

The statistical analysis did not include three children with myopia who said their overall health had “slightly deteriorated” after vision correction treatment.

Bold values indicate statistical significance (*p* < 0.05).

### 3.5 MCID development and effectiveness analysis

The MCID for the SDQ scale was established using both the distribution method and the anchoring method. Distribution analysis revealed MCID values of 2.29 and 2.38 based on 0.5 standard deviation (SD) and standard error of measurement (SEM), respectively. When using the EQ-5D-Y anxiety/depression dimension as an anchor, the MCID for SDQ difficulty scores was −2.004 when patients reported “mild improvement” (Table 5, a). Integrating these findings, this study recommends a 2-point change in SDQ difficulty total scores as the clinically important difference threshold.

**Table 5:** Development and validation of the MCID for the SDQ scale.

a. MCID developed for the SDQ scale by distributional and anchoring methods.			
MCID value	Distribution method		Anchoring method
	0.5 SD	SEM	
Total difficulties score	2.29	2.38	−2.004

b. Ratio of SDQ scale MCID to individual and group MDC at various confidence levels			
Confidence level	SDQ		
	Distributional method		Anchoring method
	0.5SD	1SEM	
95%ind	0.347	0.361	0.304
**95%group**	**2.666**	**2.771**	**2.333**
90%ind	0.415	0.431	0.363
**90%group**	**3.185**	**3.310**	**2.787**
80%ind	0.532	0.552	0.465
**80%group**	**4.082**	**4.242**	**3.572**

Bolded values indicate that MCID/MDC is greater than 1, demonstrating that MCID can reflect score changes resulting from health improvements.

To assess the validity of this MCID threshold, its ratio to the individual and group minimum detectable change (MDC) was further calculated at different confidence levels (Table 5, b). Results showed that across all calculation methods (0.5SD, 1SEM, and anchoring approach), the group-level MCID/MDC ratios exceeded 1 at 95, 90, and 80% confidence levels (e.g., 95% group-level ratios of 2.666, 2.771, and 2.333, respectively), indicating this MCID effectively identifies clinically meaningful health changes at the cohort level. However, at the individual level, the MCID/MDC ratios were less than 1 at all confidence levels (e.g., 95% individual-level ratios: 0.347, 0.361, and 0.304), suggesting this threshold is insufficient for reliably assessing clinical changes in individual patients. The MDC values at each confidence level are detailed in Supplementary Table 4.

## 4 Discussion

This is the first validation of the SDQ scale in adolescents with myopia to assess its applicability in this population. Despite limitations relied mainly on cross-sectional data dominance, which limited our exploration of the scale’s tracking validity and causal inferences, our systematic evaluation supports the SDQ’s usefulness as a quick and low-burden preliminary screening tool for mental health in pediatric myopia care, addressing the gap in population-specific instruments (28, 29). Applying the SDQ for mental health screening among myopic children facilitates rapid identification of individuals requiring further attention for psychological and behavioral issues during routine ophthalmic care, providing preliminary guidance for subsequent assessment and intervention.

The psychological and behavioral characteristics of children with myopia are unique and cannot be simply applied to the validation results of the SDQ scale for the general population (19, 30). The SDQ showed high acceptability and completion rates among myopic children, with Cronbach’s *α* and test–retest reliability (ICC) both exceeding 0.7, consistent with prior validations in general and clinical populations (31). However, the lower split-half reliability (0.532) may reflect the multidimensional nature of the SDQ and the heterogeneity of stressors in myopic children (e.g., academic pressure, peer bullying). This aligns with evidence that internal consistency (α) remains the most robust reliability metric for such complex constructs (32).

The validity analysis results revealed a strong correlation between the four difficulty subscales and the total difficulty score, indicating good content validity since each dimension’s content was homogeneous. Notably, hyperactivity/attention problems and conduct problems showed stronger correlations with total difficulties than peer problems, suggesting that difficulty subscales focusing solely on peer relationships may not fully capture impaired social functioning. Therefore, the SDQ includes advantage items in the prosocial behavior subscales to supplement the assessment. This aligns with recent research findings: school-age children affected by myopia are prone to attention disorders, and attention deficits tend to worsen as myopia progresses (21). While high subscale-total correlations generally confirmed content validity, peer-problem subscales demonstrated weaker associations. This aligns with the SDQ’s design, where prosocial behavior complements the assessment of social functioning (23). Prosocial behavior was significantly negatively correlated with peer relationship problems, hyperactivity/ inattention problems, and conduct problems. The results suggest that children with higher pro-social competence have fewer peer problems, hyperactivity/attention problems, and conduct problems. Results support prosocial behavior’s role in mitigating conduct problems (33) and suggest social activity may reduce myopia progression by limiting near-work (34).

Responsiveness was limited in the full sample, consistent with myopia’s insidious onset and minimal early functional impacts (35). This low sensitivity to short-term change underscores that the SDQ is better suited to detecting established comorbidities than acute shifts. Establishing the MCID for the SDQ in children with myopia is crucial, as it represents a key psychometric property that has not been studied previously (36). Defining the MCID aids in interpreting the practical significance of score changes, providing a basis for determining whether interventions or treatments genuinely yield clinically meaningful improvements. Recent study on health-related quality of life in children with myopia found that children with myopia are more prone to anxiety/depression problems, and the applicability of the EQ-5D-Y scale has been verified (37). In this study, MCID of 2 points—derived via distribution/anchor methods (EQ-5D-Y anxiety/depression)—proved valid for group-level monitoring (e.g., evaluating cohort interventions). However, it may lack sufficient precision for individual clinical decision-making due to measurement variability, potentially influenced by the scale’s limited number of self-rated items capturing subtle individual changes (38). Caution is warranted when applying this MCID at the individual level; it is more suitable as a reference indicator for group-level changes. Therefore, when utilizing the SDQ for mental health assessment in myopic youth, we recommend clinicians incorporate other clinical indicators (e.g., structured interviews, behavioral observations, supplementary scales) into integrated clinical judgments to enhance accuracy.

This study has several limitations: First, the sample originates solely from Shandong Province, potentially limiting the generalizability of findings to other regions. Second, the subsample size for assessing responsiveness and MCID (*n* = 59) was small, meeting only the minimum requirement (*n* ≥ 50), which may have affected the precision of parameter estimates. Additionally, our analysis lacked granularity, failing to examine differences across age groups and genders. Finally, the one-month follow-up period was too brief to assess the long-term effects of the “Double Reduction” policy on vision or to observe sustained changes in mental health.

Future research should validate findings across more diverse geographic populations. Robust validation should be conducted in larger, incorporating rules of thumb and pre-specification of efficacy analysis. More granular analyses should address demographic variations such as age and gender. Long-term follow-up studies are warranted to assess the sustained effects of the policy on vision and mental health.

## 5 Conclusion

The SDQ is a reliable and valid tool for initial screening of mental health comorbidities in children and adolescents with myopia, especially in pediatric eye care where a quick and low-burden assessment is needed. Future longitudinal studies are needed to verify the SDQ’s capacity to accurately monitor shifts in mental well-being, particularly among specific ophthalmology populations.

## Data Availability

The original contributions presented in the study are included in the article/Supplementary material, further inquiries can be directed to the corresponding author/s.

## Footnotes

1
https://www.wjx.cn/app/survey.aspx
