# Supplementary material for: Psychometric validation and determination of minimal clinically important differences for the strengths and difficulties questionnaire in adolescents with myopia
Source: Front Public Health. 2026 Jan 6;13:1730452. doi: 10.3389/fpubh.2025.1730452 (PMC12815841; doi:10.3389/fpubh.2025.1730452)
Supplement: Supplementary file 1 [file Supplementary_file_1.pdf]

Supplemental Table 1 Measurement evaluation information extraction form for psychological health research in the myopic population

Study ID	Patient population	Sample size	Country	Scale	Scale Description	Mental Health Assessment Theme	Psychometric Evaluation
J. B. Łazarczyk et al, 2016	Students	239	Poland	STAI<br>STAIC	STAI consisting of 2 subscales, 40 items<br>STAIC consisting of 2 subscales, 40 items	Anxiety	Internal consistency,<br>Content validity,
U. L. Osuagwu et al, 2023	Adults	100	Nigeria	WHOQOL-BREF<br>BDI	WHOQOL-BREF consisting of 26 items<br>BDI consisting of 21 items	Physical, Psychological, Social relationships, Environmental, and Quality of life<br>Depression	Feasibility analysis<br>Internal consistency,<br>Test-retest reliability,<br>Content validity,<br>Discriminant validity
Xiyan Zhang et al, 2024	Students	15348	China	CES-D	Consisting of 20 items	Depression	Feasibility analysis,<br>Internal consistency,<br>Content validity,<br>Responsiveness
Tae Yokoi et al, 2014	Adults	205	Japan	HADS<br>NEI-VFQ-25	HADS consisting of 2 subscales, 14 items<br>NEI-VFQ-25 consisting of 12 dimensions, 25 items	Depression and anxiety<br>Visual function, Social functioning, Mental health, Role functioning and Driving	Feasibility analysis,<br>Internal consistency,<br>Content validity,<br>Responsiveness
Yanling Yu et al, 2022	Students	6032	China	DASS-21	Consisting of 3 subscales, 21 items	Depression, anxiety, and stress	Feasibility analysis,<br>Internal consistency,<br>Content validity
Xi Xuan et al, 2021	Students	3495	China	SCL-90	Consisting of 9 subscales, 90 items	Somatization, Obsessive-compulsive symptoms, Interpersonal sensitivity, Depression, Anxiety, Hostility, Phobic anxiety, Paranoid ideation and Psychoticism	Feasibility analysis,<br>Internal consistency,<br>Content validity
Juan He et al, 2021	Students	354	China	HADS	*	*	Feasibility analysis,<br>Internal consistency
Qiuye Xu et al, 2020	Students	766	China	SAS<br>SDS	SAS consisting of 20 items<br>SDS consisting of 20 items	Anxiety；<br>Depression	Feasibility analysis,<br>Internal consistency

Dan Wang et al, 2019	Students	552	China	NEI-VFQ-25	*	*	Feasibility analysis
Xuena Wang et al, 2021	Students	504	China	SDQ	Consisting of 5 subscales, 25 items	Emotional symptoms, Conduct problems, Hyperactivity problems, Peer problems and Prosocial behavior	Feasibility analysis
Xiuwei Guo et al, 2015	Students	650	China	SCL-90	*	*	Feasibility analysis
Xiaoling Liu et al, 2012	Students	286	China	SCL-90	*	*	Feasibility analysis
Xiaoling Liu et al, 2012	Students	286	China	SCL-90	*	*	Feasibility analysis
Yuping He et al, 2016	Adults	115	China	SAS<br>SDS	*	*	Feasibility analysis
Min Zhang et al, 2015	Students	1371	China	SCL-90	*	*	Feasibility analysis
Hongyu Guan et al, 2018	Students	19934	China	MHT	Consisting of 8 dimensions, 100 items	Anxiety, Loneliness, Self-blame, Hypersensitivity, Physical symptoms, Phobic anxiety and Impulsivity	Feasibility analysis
Wen Zhang et al, 2024	Students	209	China	SCARED	Consisting of 5 dimensions, 41 items	Somatic/panic, Generalized anxiety, Separation anxiety, Social phobia and School avoidance	Feasibility analysis
Qiaoli Li et al, 2020	Students	1103	China	SAS<br>SDS	*	*	Feasibility analysis
Qiuhuan Wang et al, 2024	Students	1993	China	SCL-90	*	*	Feasibility analysis

Note: * = The scale description has been provided in preceding entries and is not repeated here

Supplemental Table 2 The frequency distribution of general information characteristics among young people

Characteristics	Group	Repeat Myopia Case (%)	First Myopia Case (%)	*P*
	**Demographic Factor**			
Gender	boy	150 (51.19)	37 (42.05)	0.132
	girl	143 (48.81)	51 (57.95)	
Age, years		10.14±2.035	9.64±1.931	0.301
Ethnicity	Han Chinese	287 (97.95)	85 (96.59)	0.461
	Others	6 (2.05)	3 (3.41)	
Education level	Primary school	213 (72.70)	78 (88.64)	**0.002**
	Middle school	80 (27.30)	10 (11.36)	
BMI	＜18	130 (44.37)	56 (63.64)	**0.005**
	18-24	131 (44.71)	28 (31.82)	
	＞24	32 (10.92)	4 (4.55)	
	**Myopia-related Factors**			
Reason for visit	Blurred vision	40 (13.65)	44 (50.00)	**<0.001**
	Screening-Detected Myopia	15 (5.12)	29 (32.95)	
	Routine ophthalmic examination	238 (81.23)	15 (17.05)	
Disease duration, years	1	193 (65.87)	/	**<0.001**
	2	41 (13.99)		
	≥3	59 (20.14)		
Myopia types	Low myopia	177 (60.41)	88 (100.00)	**<0.001**
	Moderate myopia	102 (34.81)	0 (0.00)	
	High Myopia	14 (4.78)	0 (0.00)	
Myopia management	Single vision lenses	175 (59.73)	12 (13.64)	**<0.001**
	Peripheral defocus spectacles	77 (26.28)	58 (65.91)	
	Orthokeratology lenses	21 (7.17)	13 (14.77)	
	Integrated Optical-Functional Training	20 (6.83)	5 (5.68)	
Self-reported myopia control	Good	210 (71.67)	42 (47.73)	**<0.001**
	Average	71 (24.23)	34 (38.64)	
	Poor	12 (4.10)	12 (13.64)	

Supplemental Table 3 The correlation analysis of the dimensions of the Strengths Difficulties Scale with the total score of the scale

	Prosocial Behavior	Peer Relationship Problems	Hyperactivity/ Inattention	Conduct Problems	Emotional Symptoms	Total Score
Prosocial Behavior	1.000					
Peer Relationship Problems	**-0.396*****	1.000				
Hyperactivity/ Inattention	**-0.293*****	0.137*	1.0000			
Conduct Problems	**-0.264*****	**0.217*****	**0.373*****	1.0000		
Emotional Symptoms	-0.011	0.156**	**0.297*****	**0.397*****	1.000	
Total Score	0.100	**0.358*****	**0.620*****	**0.645*****	**0.754*****	1.000

Note: **Bold values indicate** statistically significant differences (P < 0.001). * indicates *P* < 0.05; ** indicates *P* < 0.01; and *** indicates *P* < 0.001

Supplemental Table 4 The individual-level and group-level MDC of SDQ at different confidence levels

Parameter	SDQ
MDC95%	
ind	6.597
group	0.859
MDC90%	
ind	5.520
group	0.719
MDC80%	
ind	4.308
group	0.561
